# Down-regulation of the transcriptional repressor *ZNF802* (*JAZF1*) reactivates fetal hemoglobin in β^0^-thalassemia/HbE

**DOI:** 10.1038/s41598-022-08920-8

**Published:** 2022-03-23

**Authors:** Chokdee Wongborisuth, Sukanya Chumchuen, Orapan Sripichai, Usanarat Anurathaphan, Nuankanya Sathirapongsasuti, Duantida Songdej, Amornrat Tangprasittipap, Suradej Hongeng

**Affiliations:** 1 Program in Molecular Medicine, Multidisciplinary Unit, Faculty of Science, Mahidol University, Bangkok, Thailand; 2 Research Center, Faculty of Medicine Ramathibodi Hospital, Mahidol University, Bangkok, Thailand; 3 National Institute of Health, Department of Medical Sciences, Ministry of Public Health, Nonthaburi, Thailand; 4 Department of Pediatrics, Faculty of Medicine Ramathibodi Hospital, Mahidol University, Bangkok, Thailand; 5 Section of Translational Medicine, Research Center, Faculty of Medicine Ramathibodi Hospital, Mahidol University, Bangkok, Thailand

**Keywords:** Erythropoiesis, Anaemia

## Abstract

Reactivating of fetal hemoglobin (HbF; α2γ2) can ameliorate the severity of β**-**thalassemia disease by compensating for adult hemoglobin deficiency in patients. Previously, microarray analysis revealed that *zinc finger protein* (*ZNF*)*802* (also known as *Juxta-posed with another zinc finger gene-1* (*JAZF1*)) was upregulated in human erythroblasts derived from adult peripheral blood compared with fetal liver-derived cells, implying a potential role as a HbF repressor. However, deficiency in *ZNF802* induced by lentiviral shRNA in β^0^**-**thalassemia/hemoglobinE erythroblasts had no effect on erythroblast proliferation and differentiation. Remarkably, the induction of *HBG* expression was observed at the transcriptional and translational levels resulting in an increase of HbF to 35.0 ± 3.5%. Interestingly, the embryonic globin transcripts were also upregulated but the translation of embryonic globin was not detected. These results suggest ZNF802 might be a transcriptional repressor of the *γ-globin* gene in adult erythroid cells.

## Introduction

β-thalassemias and sickle cell disease are a group of inherited blood disorders caused by mutations in the *β-globin* gene cluster, which result in the reduced or absent production of β**-**globin chains of adult hemoglobin (HbA; α2β2). As a result, the relative excess of α**-**globin chains forms insoluble α**-**globin chain inclusions that cause intramedullary hemolysis and ineffective erythropoiesis^1^. Severely affected patients with β-thalassemia require lifelong blood transfusion and chelation therapy. Induction of fetal hemoglobin (HbF; α2γ2) synthesis can reduce the severity of β**-**thalassemias by improving the balance between α**-** and non**-**α**-**globin chains. The role of fetal hemoglobin in sickle cell disease was initially investigated more than 70 years ago when Janet Watson reported a few symptoms in infants with sickle cell disease who had high HbF levels in the blood^2^. Increased levels of HbF retard the polymerization of deoxy sickle hemoglobin and therefore reduce sickle hemoglobin concentrations^3^. The perinatal decline of HbF synthesis coupled with the increased synthesis of HbA occurs in the second wave of hemoglobin switching during human development, which requires the activation of several transcription factors, including those that bind to the globin gene promoters. However, the regulation of fetal to adult hemoglobin switching is not fully understood. Numerous transcription factors regulate HbF expression via silencing in definitive erythroid cells such as GATA1, KLFs, SOX6, MYB, LRF/ZBTB7A, and direct repeat erythroid-definitive (DRED) have been identified. Several studies suggested that *γ-globin* gene repression in adult cells may be regulated through the DRED complex^4^. The DRED complex is a tetrameric corepressor consisting of the orphan nuclear receptors TR2 (NR2C1), TR4 (NR2C2), and two co**-**repressor enzymes, namely DNA methyltransferase 1 (DMNT1) and lysine-specific demethylase 1 (LSD1 or KDM1a)^5^. The nuclear receptor TR4 is highly expressed in hematopoietic cells involved in the regulation of differentiation and proliferation of myeloid progenitor cells^6^. The orphan nuclear receptors have a strong binding affinity to direct repeat (DR1) elements located in the human embryonic *ε-globin* and fetal *γ-globin* promoters. Nonetheless, the human *β-globin* gene has no DR1**-**binding sites^7,8^. Inhibitory activity of the co**-**repressor enzymes, DNMT1 (by 5**-**azacytidine or decitabine) and LSD1 (by tranylcypromine or RN**-**1), led to the induction of *γ-globin* synthesis in adult definitive erythroid cells^9–11^. In addition, gene silencing of each of the DRED corepressors, *TR2*, *TR4* or *LSD1*, induced embryonic and fetal globin expression in mice^7^ and human erythroid cells^10^. These findings suggested that *γ-globin* repression in adult cells may be regulated through the DRED complex. Recently, our dataset and meta-analysis revealed a significant upregulation of *zinc finger protein* (ZNF) 802, also known as *Juxta-posed with another zinc finger gene-1* (*JAZF1*) or *TAK1-interacting protein 27* (*TIP27*) located on chromosome 7, in adult basophilic erythroblasts (CD71high/CD235a^+^)^12^. ZNF802 was reported to interact with the nuclear receptor TR4 as demonstrated by pull-down analysis^13^. We hypothesized that ZNF802 functions as a transcriptional corepressor involved in erythropoiesis and hemoglobin synthesis. Therefore, the effects of *ZNF802* knockdown were analyzed in healthy donor and β^0^**-**thalassemia/hemoglobin E (HbE) erythroblasts.

## Results

### ZNFs expression in human adult erythroblasts

**Figure 1:**
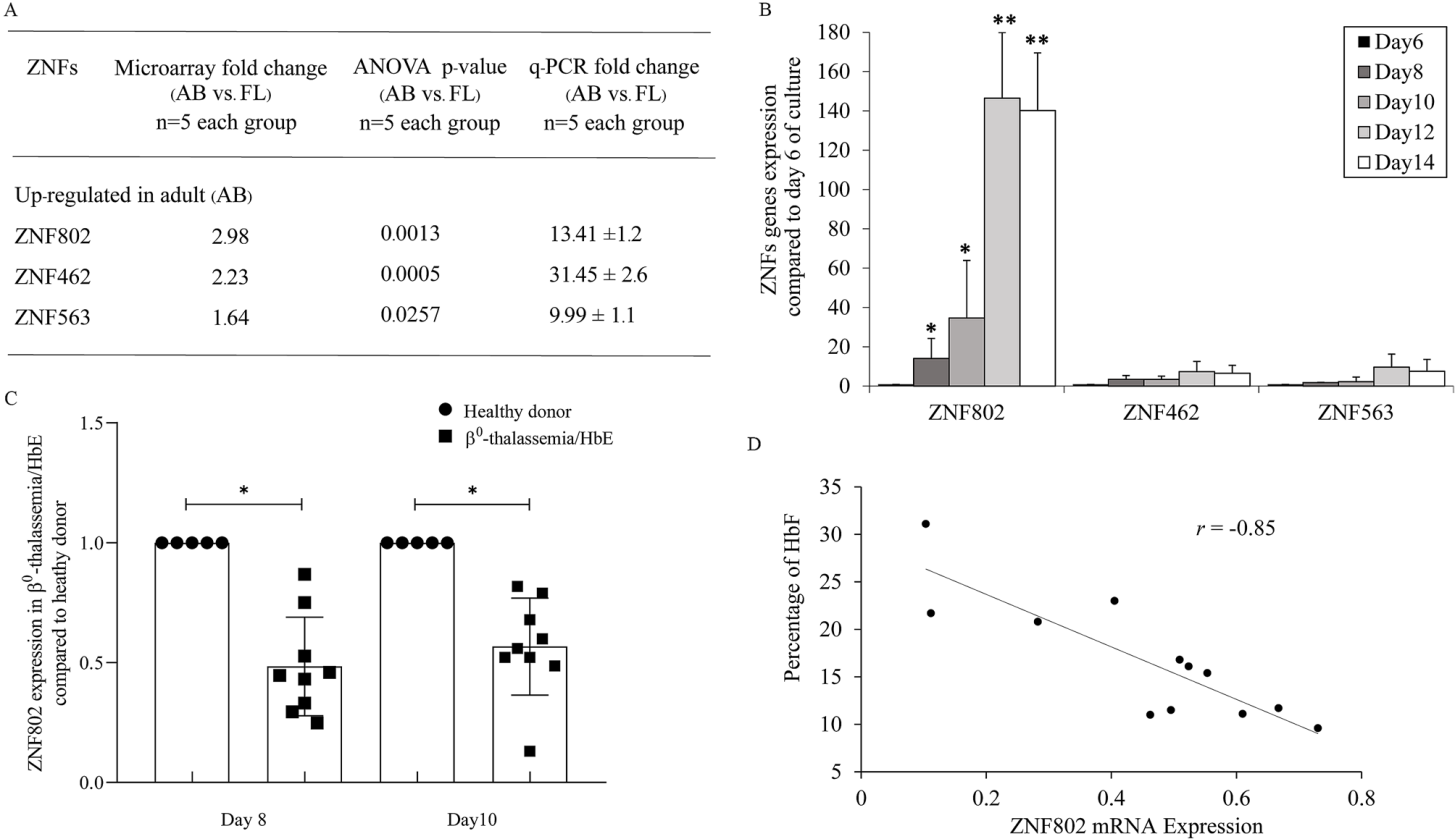
ZNF expression in human erythroid cells. (**A**) Validation of ZNF transcripts identified by microarray datasets by RT**-**qPCR. (**B**) *ZNF802*, *ZNF462,* and *ZNF563* expressions during erythroid differentiation (day 8 of culture) and the fold change analysis normalized to its expression in proerythroblasts derived from healthy donors (day 6 of culture, *n* = 3). *p < 0.05, **p < 0.005. (**C**) *ZNF802* expression at day 8 and day 10 in healthy donors (*n* = 5) and β^0^-thalassemia/HbE patients (*n* = 9). *p < 0.05. (**D**) Correlation of *ZNF802* mRNA expression at day 10 and fetal hemoglobin in β^0^-thalassemia/HbE patients (*n* = 12). Error bars represent means and ± SD.

The global gene expression profiling of stage**-**specific erythroblasts derived from fetal liver and adult peripheral blood was previously analyzed by microarray^12^. The current study further explored the transcriptional levels of 570 ZNFs in erythroblasts. The three most upregulated ZNFs in adult erythroblasts were *ZNF802*, *ZNF462*, and *ZNF563*. We validated the expression of the three most upregulated ZNFs by RT**-**qPCR using the same RNA samples as those in the microarray study. We confirmed the high expressions of three ZNFs, *ZNF802*, *ZNF462,* and *ZNF563* in adult erythroblasts compared with fetal erythroblasts (13.4**-**, 31.4**-**, and 9.9**-**folds change, respectively, Fig. 1A). In addition, three ZNF transcripts were upregulated during day 8 of adult erythroid cell differentiation when normalized to their expressions in proerythroblasts (day 6 of erythroid cell culture) derived from healthy donors (Fig. 1B). The expression of *ZNF802* showed a significant 3**–**4fold increase from day 10 to day 12 of erythroid cell differentiation. The expression patterns of *ZNF462* and *ZNF563* were slightly increased during erythroid differentiation. Interestingly, the levels of *ZNF802* expression in erythroblasts in the β^0^**-**thalassemia/HbE group (*n* = 9) cultured at day 8 and day 10 were significantly lower than in erythroblasts from healthy donors (*n* = 5; *p-*value = 0.04) (Fig. 1C) and had a strong negative correlation (r = **-**0.85; *p-*value = 0.0004) with the levels of HbF at day 14 (Fig. 1D). These results suggest that the decrease in *ZNF802* transcripts in the β^0^**-**thalassemia/HbE group indicates ZNF802 as a transcriptional repressor responsible for HbF induction in β^0^**-**thalassemia/HbE patients. Next, we investigated the effect of ZNF802 on erythropoiesis and hemoglobin production in erythroblasts.

### Knockdown of ZNF802 reactivates embryonic and fetal hemoglobins

**Figure 2:**
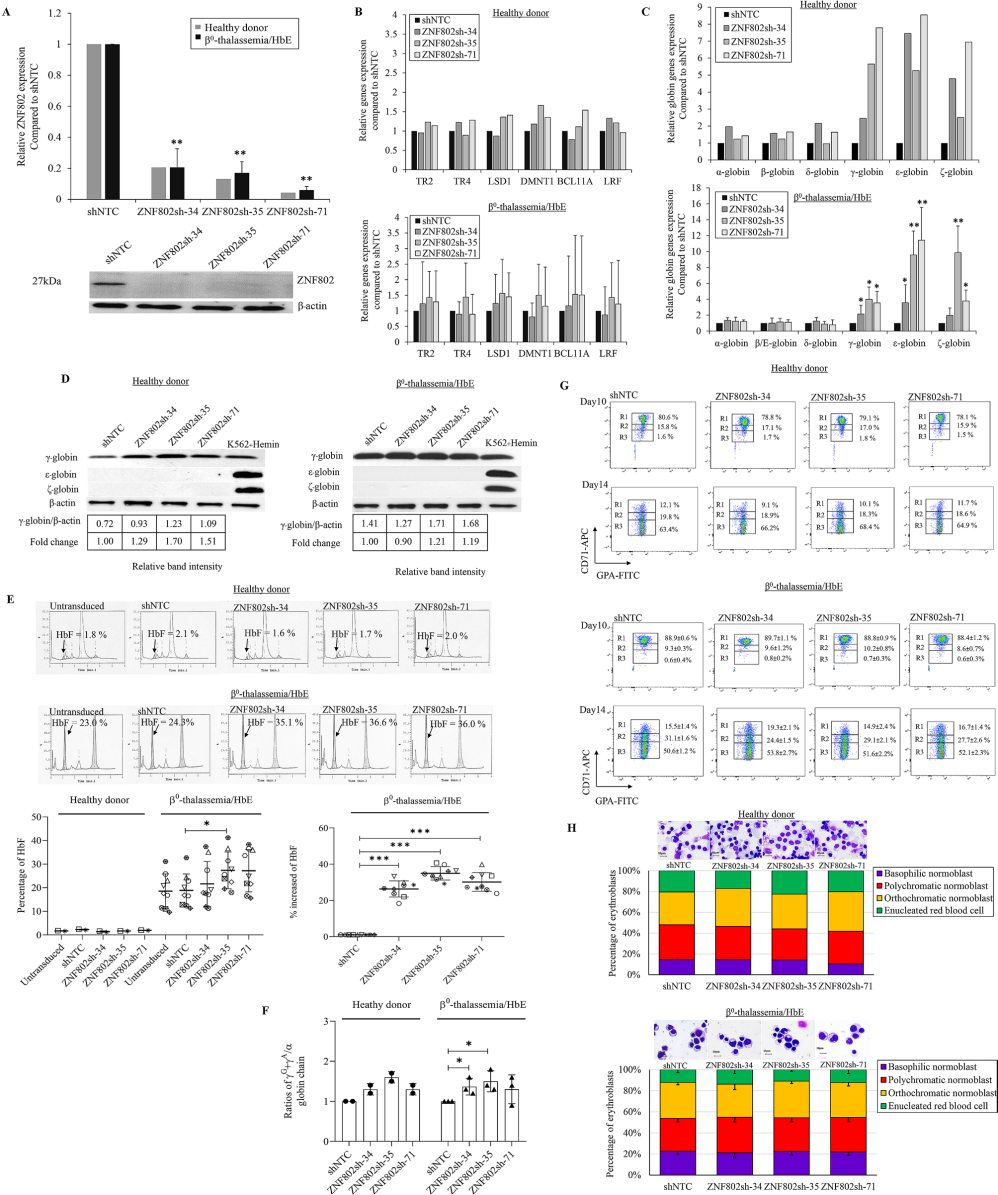
Effects of ZNF802 knockdown in erythroid progenitor cells from healthy donors (*n* = 2) and β^0^-thalassemia/HbE patients (*n* = 9). (**A**) RT-qPCR and western blotting depicting *ZNF802* knockdown efficiency on day 10. *RPS18* and β-actin were used as a housekeeping gene and loading control for RT-qPCR and western blot, respectively. (**B**) RT-qPCR of transcription factors, DRED complex repressors (*TR2*, *TR4, LSD1, DMNT1*), major repressor *BCL11A,* and *LRF* mRNA expression levels on day 10. *p < 0.05, **p < 0.005. (**C**) RT-qPCR analysis of the relative fold change of *α-globin* and *β- globin* cluster mRNA expression levels. Relative fold change represents the mRNA expression levels normalized to *RPS18* of shRNAs targeting *ZNF802* (ZNF802sh-34, ZNF802sh-35, and ZNF802sh-71) versus nontargeting control shRNA (shNTC) on day 10. Data are presented as the mean ± SD from healthy donors and β^0^-thalassemia/HbE patients, *p < 0.05. (**D**) Representative western blot analysis on day 10 showing each globin protein expression. β-actin was used as a loading control. (**E**) Illustrative hemoglobin typing showing HbF induction on day 14, *p < 0.05. Percentage increase of HbF compared with shNTC, ***p < 0.001. (**F**) Globin chain analysis to determine the relative ratio of γ-globin chain induction in healthy donors (*n* = 2) and β^0^-thalassemia/HbE patients (*n* = 3), *p < 0.05. (**G**). Illustrative flow cytometric analysis on day 10 and day 14 showing transferrin receptor (CD71) and glycophorin A (GPA) expression levels. R1 population, CD71high/GPAhigh; R2 population, CD71medium/GPAhigh; R3 population, CD71low/GPAhigh. (**H**) Representative cytospin preparations of modified Giemsa-stained cells on day 10 from healthy donors (*n* = 2) and β^0^-thalassemia/HbE patients (*n* = 5) visualized under a light microscope with 100 × magnification. Scale bar indicates 10 μm. Error bars represent means and ± SD.

The expression of *ZNF802* was knocked down in adult erythroid progenitor cells to investigate the potential role of ZNF802 as a transcriptional repressor of HbF synthesis in adult erythroid cells. The expression levels of six human globin genes, *HBZ*, *HBE*, *HBG*, *HBA*, *HBD,* and *HBB* were determined by RT**-**qPCR after *ZNF802* knockdown in human erythroid progenitor cells from healthy donors and β^0^**-**thalassemia/HbE subjects. Lentiviral vectors carrying three specific ZNF802 shRNAs, including ZNF802sh**-**34, ZNF802sh**-**35, and ZNF802sh**-**71, reduced *ZNF802* transcripts by more than 80% (Fig. 2A) and resulted in an almost undetectable protein level of ZNF802 in erythroblasts compared with the non**-**targeting control shRNA group (shNTC) (Fig. 2A). Knockdown of *ZNF802* did not significantly affect the expressions of the major HbF repressors *BCL11A* and *LRF*, or DRED complex members including *TR4*, *TR2*, *LSD1*, and *DMNT1* (Fig. 2B), which demonstrated the specificity of the ZNF802 shRNAs.

We observed a significant upregulation of the embryonic transcripts; *HBZ* 7-fold change and 10-fold change, *HBE* 8- fold change and ** 12-**fold change, and fetal transcripts; *HBG* 8-fold change and 4-fold change of mRNA expression in *ZNF802* knockdown cells compared with shNTC cells at day 10 in healthy donors and β^0^**-**thalassemia/HbE cases, respectively (Fig. 2C). The expression of globin gene transcripts in ZNF802sh**-**34, ZNF802sh**-**35, and ZNF802sh**-**71 knockdown erythroblast was reported as fold change compared with its expression derived from shNTC. When *ZNF802* expression was diminished in erythroblasts derived from a healthy donor, the *HBA*, *HBB* and *HBD* transcript were expressed in the range of 1.2**–**1.9 fold change, 1.2**–**1.6 fold change and 0.9**–**2.1 fold change, respectively. In β^0^**-**thalassemia/HbE, the *HBA*, *HBB* and *HBD* transcripts were expressed in the range of 1.2**–**1.3 fold change, 0.2**–**1.1 fold change and 0.8**–**1.3 fold change, respectively. The *HBA*, *HBD*, and *HBB* transcripts were consistently expressed after ZNF802 shRNA transduction by lentiviral vector. Western blot analysis demonstrated that only the levels of γ**-**globin protein post ZNF802**-**sh35 knockdown were increased (1.7**-** and 1.2**-**fold changes in healthy donors and β^0^**-**thalassemia/HbE, respectively), whereas the embryonic globin (HBZ, HBE) proteins were undetectable in erythroblasts transduced with ZNF802**-**shRNA (Fig. 2D). Knocking down the expression of *ZNF802* by ZNF802sh**-**35 and ZNF802sh**-**71 in β^0^-thalassemia/HbE erythroid cells reactivated HbF to 26.9 ± 7.4% and 26.4 ± 8.8%, respectively, whereas the baseline HbF levels of shNTC varied between 12**–**23% with a mean of 18.5 ± 6.8%, (*n* = 9, Fig. 2E). Therefore, the increase in the percentage of fetal hemoglobin (% increase of HbF) was analyzed by the ratio of HbF in the ZNF802 knockdown compared with the shNTC in individual β^0^**-**thalassemia/HbE samples. The percentage of HbF was significantly increased to 26.3 ± 4.2% in the ZNF802sh**-**34 group, 35.0 ± 3.5% in the ZNF802sh**-**35 group, and 30.2 ± 4.8% in the ZNF802sh**-**71 group compared with the shNTC of β^0^**-**thalassemia/HbE (p**-**value < 0.0001, Fig. 2E). The increase in γ**-**globin chain levels in *ZNF802* knockdown erythroblasts was demonstrated by the relative ratio γ to α-globin chain (γA + γG/α**-**globin chain) compared with shNTC treatment in the same donor (Fig. 2F). These changes in HbF levels were concomitant with a significant increase in *γ-globin* transcripts and γ**-**globin chain analysis by HPLC (Figs. S1, 2F). Erythroid differentiation and maturation were analyzed based on the expressions of CD71 and GPA, including the CD71**-**high/GPA**-**high population (R1), the CD71**-**medium/GPA**-**high population (R2), and the CD71**-**low/GPA**-**high population (R3). In this culture system, β^0^**-**thalassemia/HbE**-**derived erythroblasts demonstrated delayed erythroid differentiation (greater R1 portion) compared with those from healthy donors (Fig. 2G). The downregulation of *ZNF802* in erythroblasts derived from healthy donors and the β^0^**-**thalassemia/HbE group did not affect erythroid differentiation or maturation as visualized by flow cytometry and Giemsa staining (Figs. 2G, H).

## Discussion

The phenomenon of hemoglobin switching is mediated by transcriptional changes in hemoglobin composition (embryonic**-**to fetal**-**to adult**-**globin) at different developmental stages^14^. This process has been studied intensively because the reactivation of HbF can ameliorate the clinical symptoms of β**-**hemoglobinopathies. However, the mechanisms underlying the switching remain unclear. The critical switching regulators of fetal to adult globin gene expression potentially involve DNA-binding TFs *BCL11a* and *LRF* (*ZBTB7A*) and the nucleosome remodeling and deacetylase (NuRD) chromatin complex^15–17^. Zinc-finger TFs, *BCL11a* and *LRF* bind to unique site at the proximal promoters of the duplicated gene *HBG1* and *HBG2* and interact with NuRD to silence γ-globin transcription^16,18^. The general mechanism allowing the reactivation of HbF levels is through the inactivation by any silencer or repressor (s) of fetal globin expression in adult erythroid cells. However, the reactivation HbF by inactivated HbF silencers such as *BCL11a* and *LRF* may negatively contribute to various hematopoietic lineages^19,20^ and terminal erythropoiesis^21^, respectively. Therefore, new target genes or drugs that can induce HbF with less or no adverse effects are highly needed. In this study, we took advantage of our previous microarray analysis of the comparative gene expression profiling of human erythroid cells derived from the fetal liver and adult peripheral blood. We explored the expression of 570 ZNFs in erythroblasts^12^. ZNFs contain a zinc**-**finger domain that typically binds to DNA, RNA, and proteins involved in several cellular processes, which mediate their effects through different molecular mechanisms in eukaryotes^22^. Among the significantly differentially expressed ZNF transcripts, the upregulation of *ZNF802* in adult erythroid cells was confirmed by RT-qPCR. In addition, the level of *ZNF802* expression in erythroblasts (day 8) revealed a negative correlation with HbF levels in β^0^**-**thalassemia/HbE erythroid cells, which support a repressive role for *ZNF802* in HbF production (day 14). Recently, a genome-wide association study of an African American cohort with sickle cell disease reported a single nucleotide polymorphism (SNP) of *ZNF802*/*JAZF1* (SNP ID = rs740127, base position 28,004,900) associated with high HbF levels with a low genome-wide significance^23^. This evidence supports our assumption that ZNF802 may have an important role of hemoglobin production during adult erythropoiesis.

To investigate whether *ZNF802* has a role in globin regulation related to diverse HbF baseline levels, we performed lentiviral shRNA**-**mediated *ZNF802* knockdown in erythroid progenitor cells from healthy donor adults and β^0^**-**thalassemia/HbE patients. We successfully knocked down *ZNF802* expression, which resulted in the induction of embryonic globin *(HBZ* and *HBE*) and fetal globin (*HBG*) gene expressions in erythroid progenitor cells from healthy donors and β^0^**-**thalassemia/HbE subjects, similar to the increase in embryonic *εy* and *βh1* globins when the DRED complex partnership, *TR2* and *TR4*, was knocked out in mouse erythroid cells^24^. The upregulation of embryonic and fetal globin gene expression in *ZNF802* knockdown erythroid cells supported our assumption that ZNF802 might function as a corepressor related to TR4 binding at the DR**-**1 binding site of these globin promoters^13^. The diminished *ZNF802* may perturb the binding affinity of TR4 on the DR1 element, resulted in *γ-globin* and *ε-globin* mRNA induction. The reactivation of *γ-globin* expression could bind to excess α**-**globin in β^0^**-**thalassemia/HbE cells, which do not have adult hemoglobin, then formed tetrameric of HbF. In comparison, the assumption may not happen in healthy donors who have balance of β/α**-** globin ratio. Interestingly, the downregulation of *ZNF802* did not significantly affect the expression levels of major repressors of *HBG* including *BCL11A* and *LRF*, or other transcription factors of the DRED complex, suggesting an independent mechanism of globin regulation. We did not detect the protein levels of embryonic globins by western blotting or globin chain analysis via HPLC. This might be because the transcripts of embryonic globin were not translated into proteins or the levels of embryonic globin chains were lower than the limit of detection. Commonly, the basal level of HbF in peripheral blood derived from healthy donors is very low (1–3% HbF). The γ**-**globin protein was increased 1.7-fold by diminishing *ZNF802* compared with shNTC in healthy donors, which might have been related to the slight increase in HbF. Conversely, *γ-globin* expression was upregulated 1.2**-**fold when *ZNF802* was knocked down in erythroblasts from β^0^**-**thalassemia/HbE patients, as reflected by the transcriptional and translational levels of HbF compared with those in the shNTC group in terms of % increased HbF. In this study, we focus on the β^0^**-**thalassemia/HbE group with a wide range of HbF levels (9.6–31.1%) to study the diminishing effect of *ZNF802* on fetal hemoglobin regulation. The diminished *ZNF802* expression in sickle erythroblasts or other β**-**hemoglobinopathies may reactivate the *γ-globin* expression via the DRED complex and increase HbF levels, but this needs to be verified further study. However, the diminishing effect of *ZNF802* in some groups of β**-**thalassemia like homozygous β^+^**-**thalassemia and homozygous β^0^**-**thalassemia who a high levels of fetal hemoglobin (70**–**90%)^25^, may pose some difficulties in distinguishing the upregulation of *γ-globin* expression and HbF production.

Recently, ZNF410 (pentadeacytl ZF protein) has emerged as a DNA**-**binding protein that directly interacts with *CHD4,* as shown by CRISPR-Cas9 screening^26,27^. Loss of ZNF410 in the adult stage of erythroid cell culture systems and xenotransplantation diminishes *CHD4* levels and derepresses the fetal hemoglobin genes^26^. In addition, knocking down of *ZNF410* demonstrated no defects on erythroid maturation or hematopoietic reconstitution^27^. *ZNF410* is a novel HbF repressor that does not directly bind to HBG promoter regions but it acts specifically to enhance the expression of the CHD4 component of the NuRD complex. The discovery of *ZNF410* demonstrated a new repressor that regulated γ-globin via the CHD4 component of the NuRD complex. Conversely, the DRED complex corepressor is one of the critical factors for γ**-**globin repressors in adult erythropoiesis^4^. We speculate that ZNF802 may be a member of the DRED complex that interacts with TR4 and binds on the ε**-** and γ**-** promoter regions of DR1. The transcriptomic profile derived from knocking down *ZNF802* in erythroid cells may involve the responsive target gene cluster that reactivates *γ-globin* and *ε-globin,* but the RNA derived from *ZNF802* knockdown erythroid cells was not qualified for RNAseq. The lacking information on DNA**-**binding of ZNF802 hampered the attempt to determine the mechanism of how *ZNF802* regulates HBG gene expression, while chromatin immunoprecipitation (ChIP) revealed no suitable candidate antibody^28^.

Epigenetic factors might contribute to the modifying of the chromatin structure of globin genes (and eventually other genes) and be responsible for regulating globin gene expression and disease severity. Recently, ZNF802 was suggested to be a chromatin modulator that recruits components of the histone H2A.Z chaperone complex in regulatory regions to control gene expression^29^. Incorporating of histone H2A.Z into chromatin might have allowed enhancers more access by transcription factors or coactivators before transcription initiation. H2A.Z was highly enriched on active enhancers or locus control regions (LCR HSs; HS4, HS2, HS1) of the *β-globin* locus, either the active *γ-globin* promoter of the erythroleukemic cell line, K562, or the active *β-globin* promoter of murine erythroleukemia cells^30^, suggesting its role in chromatin reorganization during erythropoiesis and hemoglobin production. H2A.Z incorporation facilities RNA pol II elongation^31^. Disrupting the expression of *ZNF802* in erythroid cells by shRNA may be associated with different epigenetic regulatory mechanisms, including the deacetylation of H2A.Z on LCR or adult *β-globin* gene promoters. ZNF802 may function in combination with other repressors of the DRED complex by binding at the *γ-globin* promoter or serve as a chaperone partner with histone H2A.Z at LCR or *γ-globin* promoters. In this context, we speculate that ZNF802 represses the *ɛ-* and *γ-globin* genes via the interaction with TR4 component of the DRED complex binding to the DR1 region of *ε- globin* and *γ-globin* promoter and the potentially modulate the histone modification of H2A.Z at specific LCR hypersensitive sites and promoters of active globin genes. Further investigation should elucidate the role of ZNF802 in chromatin remodeling at LCR looping to the *γ-globin* or *β-*globin promoters on β**-**clusters.

## Methods

### Subjects and sample collection

This study was performed after obtaining institutional ethical approval (MURA2017/375) from the Ethics Committee on Human Rights Related to Research Involving Human Subjects, Faculty of Medicine Ramathibodi Hospital, Mahidol University, Thailand. Mobilized peripheral blood progenitor cells were collected by leukapheresis from healthy donors. Bone marrow samples were obtained as part of pre-stem cell transplantation back up from β^0^**-**thalassemia/HbE patients at the Department of Pediatrics, Faculty of Medicine Ramathibodi Hospital. Each participating subject provided written informed consent and all experiments were performed in following relevant guidelines and regulations.

### Isolation of hematopoietic stem cells

Mononuclear cells were separated from the mobilized peripheral blood of healthy donors and bone marrow from β^0^**-**thalassemia/HbE patients by the gradient density centrifugation (1.077 g/mL Lymphoprep®, Axis-Shield PoC AS, Oslo, Norway) and subsequently selected for CD34^+^ cells using positive immunomagnetic selection (CD34 MicroBead Kit) (Miltenyi Biotec, Bergisch Gladbach, Germany) according to the manufacturer’s recommendations.

### Erythroid differentiation of CD34^+^ cells

Purified CD34^+^ cells were cultured and differentiated ex vivo into the erythroid lineage using the two-phase culture method. Cells were cultured for 4 days in phase I medium consisting of Iscove’s Modified Dulbecco’s Medium (IMDM; Gibco, Grand Island, NY, USA) supplemented with 20% of fetal bovine serum (FBS; Sigma**-**Aldrich, St**-**Louis, MO, USA), 300 μg/mL of holo**-**transferrin (holo**-**TF; PromoCell, Heidelberg, Germany), 10 ng/mL of interleukin-3 (IL**-**3; Cell Signaling Technologies, Beverly, MA, USA), 50 ng/mL of human stem cell factor (SCF; Cell Signaling Technologies, Beverly, MA, USA), and 2 units/mL of human recombinant erythropoietin (EPO; CILAG GmbH, Zug, Switzerland). After 4 days, suspended cells were collected and re-seeded in phase II medium consisting of IMDM supplemented with 20% of FBS, 300 μg/mL of holo**-**TF, and 5 units/mL of EPO. The culture was maintained under an atmosphere of 5% CO_2_ at 37 °C for 10 days in phase II medium. Erythroblast differentiation was monitored by cell surface marker analysis using flow cytometry on a FACSVerse flow cytometer (BD Biosciences, San Jose, CA, USA), in which cells were immunostained with allophycocyanin**-**conjugated anti**-**transferrin receptor (CD71-APC) (BD Biosciences Pharmingen, San Diego, CA, USA) and fluorescein isothiocyanate-conjugated anti-glycophorin A (GPA-FITC) (BioLegend, San Diego, CA, USA) antibodies. In addition, cell maturation was monitored by Giemsa**-**stained cytospin preparations. Cell morphology was observed under a light microscope.

### Lentiviral shRNA-ZNF802 production and titer determination, and ZNF802 knockdown

shRNAs targeting human ZNF802 mRNA were obtained from the Broad Institute Genetic Perturbation Platform Web Portal and Sigma Aldrich. Three selected target shRNA sequences (shRNA-TRCN0000078534, shRNA-TRCN0000078535, and shRNA-TRCN0000256871) were cloned into the third-generation lentiviral vector, pLL3.7-puro, which was an in-house modified form of pLL3.7 (Addgene plasmid #11795) that replaced the EGFP gene with a puromycin resistance gene as a selectable marker and containing the mouse U6 promoter to drive shRNA expression (a gift from Dr. Khamphikham)^32^. Lentiviruses expressing different shRNAs targeting ZNF802 mRNA were produced by co-transfecting 10 μg of the expression constructs with packaging plasmids including 2.5 μg of pMD2.G (Addgene plasmid #12259), 3.75 μg of pMDLg/pRRE (Addgene plasmid #12,251), and 3.75 μg of pRSV-Rev (Addgene plasmid #12253) into HEK293T cells using X-tremeGENE HP Transfection reagent (Roche Molecular Systems, CA, USA). Supernatants were collected at 48 and 72 h after transfection and were filtered through a 0.45-μm membrane. The filtrates were concentrated using a Lenti-X concentrator (Clontech, Mountain View, CA, USA) and centrifuged at 1500 × g at 4 °C for 1 h. Lentiviral titers were measured by crystal violet staining (https://horizondiscovery.com/-/media/Files/Horizon/resources/Protocols/titer-crystal-violet-protocol.pdf). Briefly, HEK293T cells were transduced with serial dilutions of lentiviral carrying shRNA in the presence of 4.0 μg/mL polybrene (Sigma-Aldrich), and subsequently challenged with 2.0 μg/mL puromycin (Invitrogen, Carlsbad, CA, USA) at 48 h post-transduction then stained with 1% crystal violet in 10% ethanol. Transducing units per ml (TU/mL) were calculated by colony counts per volume (mL) multiplied by the dilution rate. A non-targeting control shRNA sequence (SHC016V; Sigma-Aldrich) was used as a negative control (shNTC). Day 4 erythroblast cells in culture were transduced with the lentiviruses at a multiplicity of infection (MOI) of 20 in phase II medium supplemented with 8.0 μg/mL polybrene overnight. The transduced cells were treated with 1.0 μg/mL puromycin for 48 h post-transduction, and then cultured in phase II medium (without puromycin) until day 14.

### RNA isolation and reverse-transcription quantitative PCR (RT-qPCR)

Total RNA was extracted from erythroid cultures (2 × 10^6^ cells) using TRIzol Reagent (Thermo Fisher Scientific, MA, USA) according to the manufacturer’s instructions. cDNA was synthesized by reverse transcriptase reaction using the RevertAid First Strand cDNA synthesis kit (Thermo Fisher Scientific), following the manufacturer’s protocol. RT-qPCR was performed in duplicate with the specific primers listed in Table S1 using FastStart Essential DNA Green Master Mix (Roche Diagnostics, CA, USA) and analyzed by a LightCycler® 96 System (Roche Molecular Systems). All target gene expression levels were normalized to ribosomal RNA S18 (RPS18). The relative fold change was analyzed using the 2^-∆∆Ct^ method^33^.

### Western blotting

Nuclear and cytoplasmic proteins were extracted from a pellet of at least 5 × 10^6^ cultured cells on day 10 using NE-PER, Nuclear and Cytoplasmic Extraction Reagents (Thermo Fisher Scientific) following the manufacturer’s instructions. Protein concentration was determined by the Bradford Protein Assay (BioRad, CA, USA). Ten micrograms of nuclear extract protein was run on a 12% SDS polyacrylamide gel, transferred to a polyvinylidene fluoride membrane, and blocked with 5% skimmed milk in phosphate buffer saline supplemented with 0.05% Tween 20 (PBST) (Sigma-Aldrich) for 1 h. Immunoblotting was performed using specific antibodies against their target proteins (Table S1) overnight at 4 °C. The membrane was washed three times for 10 min each with PBST. HRP-conjugated secondary antibodies were used to probe at room temperature for 1 h, followed by three times wash before signal development. Chemiluminescent detection was carried out using ECL™ western blotting detection reagents (Thermo Fisher Scientific) and detected by exposure to an X-ray film.

### Hemoglobin typing

Hemolysates were prepared from at least 1 × 10^6^ cultured cells on day 14 in VAR-β-THAL Elution buffer 1 (BioRad) and used for high-performance liquid chromatography (HPLC) for hemoglobin type analysis using a Bio-Rad VARIANT II Hemoglobin Testing System with β-Thalassemia Short Program (BioRad) according to the manufacturer’s recommendations.

### Globin chain analysis by high-performance liquid chromatography

Cells were lysed in HPLC-quality distilled water and then underwent two freeze–thaw cycles. A clear cell lysate was separated by centrifugation at 14,000 × *g* for 10 min at 4 °C and the supernatant was transferred into an HPLC micro vial. Analyzes were performed on a Waters HPLC alliance e2695 (Waters Corporation, MA, USA) separations module and detector. The stationary phase was collected on an Aeris 3.6-µm WIDEPORE-C4 200 Å column behind a SecurityGuard UHPLC Wide-pore C18; 4.6 mm guard column (Phenomenex, CA, USA). The mobile phase was composed of buffer A, 0.1% trifluoroacetic acid (Sigma-Aldrich), in deionized water and buffer B, 0.1% trifluoroacetic acid in 95% acetonitrile (E-CHROMASOLV for HPLC; Sigma Aldrich). At the start of each sample injection, the ratio of mobile phase Buffer A and Buffer B was 60:40%. Buffer B was gradually increased to 53% after 55 min with a constant flow rate of 1 mL/min. The eluted globin proteins were measured at 220 nm with a UV detector (photodiode array detector). Empower 3 chromatography software was used for data acquisition and data analysis.

### Statistical analysis

All statistical analyses were performed using an unpaired Student’s *t*-test and Prism 8 version 8.4.3 (GraphPad Software, San Diego, CA). Results are presented as the mean ± SD and *p*-values < 0.05 were considered significant.

### Ethics declarations

This study was performed after obtaining institutional ethical approval (MURA2017/375) from the Ethics Committee on Human Rights Related to Research Involving Human Subjects, Faculty of Medicine Ramathibodi Hospital, Mahidol University, Thailand.

## Supplementary Information

Supplementary Information.
