# Author Correction: Down-regulation of the transcriptional repressor *ZNF802* (*JAZF1*) reactivates fetal hemoglobin in β^0^-thalassemia/HbE

**DOI:** 10.1038/s41598-022-10541-0

**Published:** 2022-04-13

**Authors:** Chokdee Wongborisuth, Sukanya Chumchuen, Orapan Sripichai, Usanarat Anurathaphan, Nuankanya Sathirapongsasuti, Duantida Songdej, Amornrat Tangprasittipap, Suradej Hongeng

**Affiliations:** 1 Program in Molecular Medicine, Multidisciplinary Unit, Faculty of Science, Mahidol University, Bangkok, Thailand; 2 Research Center, Faculty of Medicine Ramathibodi Hospital, Mahidol University, Bangkok, Thailand; 3 National Institute of Health, Department of Medical Sciences, Ministry of Public Health, Nonthaburi, Thailand; 4 Department of Pediatrics, Faculty of Medicine Ramathibodi Hospital, Mahidol University, Bangkok, Thailand; 5 Section of Translational Medicine, Research Center, Faculty of Medicine Ramathibodi Hospital, Mahidol University, Bangkok, Thailand

Correction to: *Scientific Reports* 10.1038/s41598-022-08920-8, published online 23 March 2022

The original version of this Article contained an error in the spelling of the author Usanarat Anurathaphan which was incorrectly given as Anurathaphan Usanarat. The original Article has been corrected.

