# Supplementary material for: Down-regulation of the transcriptional repressor ZNF802 (JAZF1) reactivates fetal hemoglobin in β0-thalassemia/HbE
Source: Sci Rep. 2022 Mar 23;12:4952. doi: 10.1038/s41598-022-08920-8 (PMC8943019; doi:10.1038/s41598-022-08920-8)
Supplement: Supplementary file 1 — Supplementary Information. [file 41598_2022_8920_MOESM1_ESM.pdf]

Down-regulation of the transcriptional repressor *ZNF802* (*JAZF1*) reactivates fetal hemoglobin in β^0-thalassemia/HbE

Chokdee Wongborisuth[1,2], Sukanya Chumchuen[2], Orapan Sripichai[3], Usanarat Anurathapan[4], Nuankanya Sathirapongsasuti[5], Duantida Songdej[4], Amornrat Tangprasittipap[2,*], and Suradej Hongeng[4,*]

[1]Program in Molecular Medicine, Multidisciplinary Unit, Faculty of Science, Mahidol University, Bangkok, Thailand

[2]Research Center, Faculty of Medicine Ramathibodi Hospital, Mahidol University, Bangkok, Thailand

[3]National Institute of Health, Department of Medical Sciences, Ministry of Public Health, Nonthaburi, Thailand

[4]Department of Pediatrics, Faculty of Medicine Ramathibodi Hospital, Mahidol University, Bangkok, Thailand

[5]Section of Translational Medicine, Research Center, Faculty of Medicine Ramathibodi Hospital, Mahidol University, Bangkok, Thailand

*Correspondence: tamornrat01@gmail.com

Supplementary information

Genes	Forward primer (5' to 3')	Reverse primer (5' to 3')
ZNF802	AGCTCCATGTGCATGAATGG	TTGAATGGTTTGCGGACACG
ZNF462	TTTTGTGGACGGGCGTTTTC	ATTCAATGCCATGCCGTGTC
ZNF563	AGAAACCATCAGGAACCTGGAC	GTTTCTCCACACTGACTACTGTC
α-globin	TGGACCCGGTCAACTTCAAG	TCACAGAAGCCAGGAACTTGTC
β-globin	GAAGGCTCATGGCAAGAAAG	CACTGGTGGGGTGAATTCTT
δ-globin	CAAAGTGAACGTGGATGCAG	CTGAGAAAAAGTGCCCTTGAG
γ-globin	TCACAGAGGAGGACAAGGCTA	GCTTTATGGCATCTCCCAAG
ε-globin	GAATGTGGAAGAGGCTGGAG	GGCTTGAGGTTGTCCATGTT
ζ-globin	TGAGAGGACCATCATTGTGTCC	AAGTGCGGGAAGTAGGTCTTG
RPS18	GGATGAGGTGGAACGTGT	CTAGGACCTGGCTGTATTTTC
TR4	TTGTGAAGGTTGCAAAGGTT	TTCATGCCCATCTCTAAGCA
TR2	GGAGACAAAGCATCAGGACG	ATCTCTGTAACCTGCAGTAT
LSD1	AATTATTATAGGCTCTGGGG	TTCCCTCCAAGACCTGTTAC
DMNT1	GACAGAGAAGACAAGGAGAA	TATCATCGACTTCCTCATCG
LRF	CTTCACCAGGCAGGACAA	GGTTCTTCAGGTCGTAGTTGTG
BCL11A	GGGAATTCTCGCCCGAG	GGGAAGTTCATCTGGCACT

Table S1. List of primers for real time PCR.

Antibodies	Species specificity	Type	Dilution Rate	Manufacturer	Cat no.
JAZF1	rabbit anti-human	polyclonal antibody	1:10,000	Abcam	ab199791
Hemoglobin-γ	mouse anti-human	monoclonal antibody	1:2,000	Santa Cruz Biotechnology	sc-21756
Hemoglobin-ε	rabbit anti-human	polyclonal antibody	1:1,000	Abcam	ab156041
Hemoglobin-ζ	rabbit anti-human	polyclonal antibody	1:5,000	Abcam	ab62266
Secondary antibody	goat anti-rabbit	IgG conjugated with HRP	1:10,000	Abcam	ab97051
Secondary antibody	goat anti-mouse	IgG conjugated with HRP	1:10,000	Cell Signaling	#7076S
Beta actin	mouse anti-human	monoclonal antibody conjugated with HRP	1:20,000	Abcam	ab49900

Table S2. List of antibodies for immunoblotting.

	Untransduced	shNTC	ZNF802-sh34	ZNF802-sh35	ZNF802-sh71
Healthy donor 1	1.8 %	2.1 %	1.6 %	1.7 %	2.0 %
Healthy donor 2	1.6 %	2.4 %	1.2 %	1.6 %	1.9 %
β-thal/HbE 1	9.6 %	11.3 %	11.9 %	19.5 %	15.6 %
β-thal/HbE 2	11.5%	12.4 %	17.6 %	18.0 %	16.5 %
β-thal/HbE 3	11 %	12.8 %	11.3 %	23.8 %	21.8 %
β-thal/HbE 4	16.8 %	16.5 %	17.0%	31.4 %	33.6 %
β-thal/HbE 5	25.8 %	22.9 %	27.4 %	25.5 %	36.5 %
β-thal/HbE 6	17.6 %	16.8 %	17.9 %	22.4 %	21.6 %
β-thal/HbE 7	20.8 %	20.1 %	19.1 %	27.8 %	24.8 %
β-thal/HbE 8	23.0 %	24.3 %	35.1 %	36.6 %	36.0 %
β-thal/HbE 9	31.1 %	32.7 %	37.4 %	41.2%	38.3 %

Table S3. Percentage of HbF of healthy donors and β^0 Thalassemia/HbE patients.

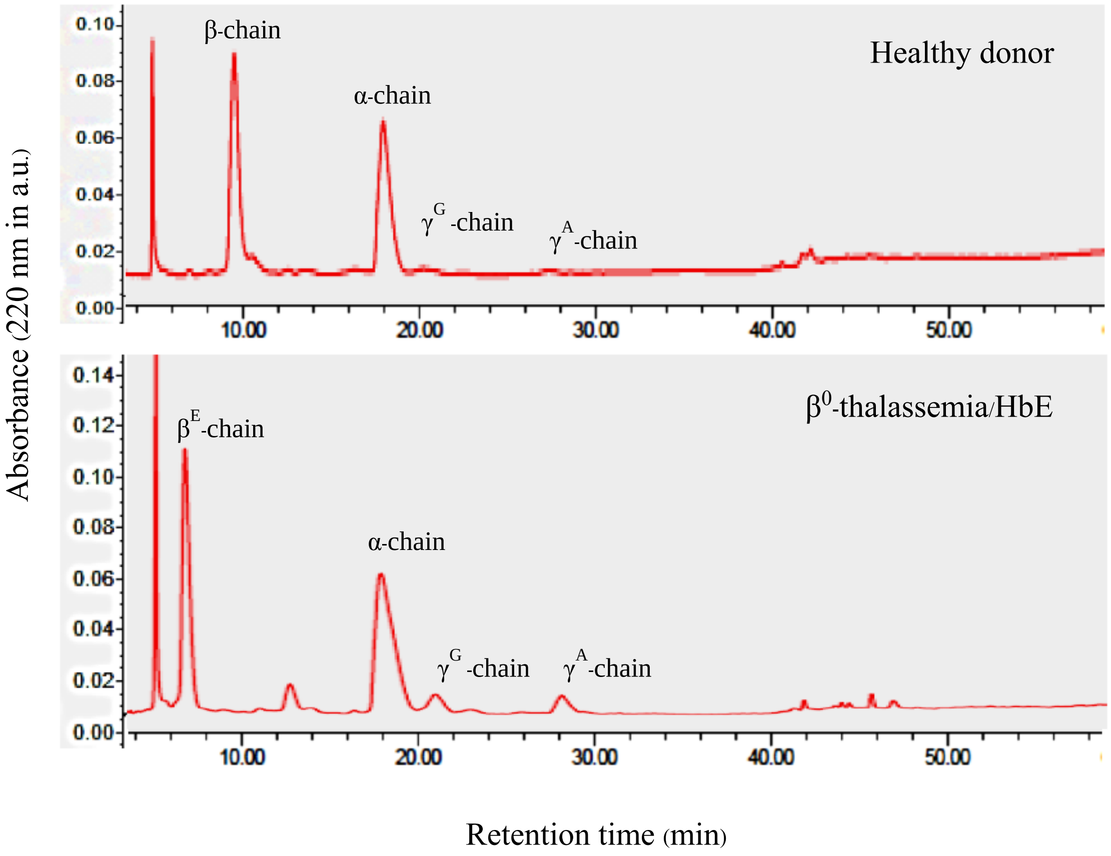

Percentage of globin chains (% area)								
	Healthy donor (n=2)				β^0-thalassemia/HbE (n=3)			
	α-globin	β-globin	$^G\gamma$-globin	$^A\gamma$-globin	α-globin	β^E-globin	$^G\gamma$-globin	$^A\gamma$-globin
Untransduced	49.2	48.9	1.2	0.6	53.3±0.5	37.6±2.2	5.7±2.3	3.4±0.6
shNTC	49.7	47.6	1.75	0.8	53.5± 1.7	36.1± 3.2	6.5±2.5	3.8±0.5
ZNF802sh-34	48.6	47.6	2.45	1.3	53.4±1.4	33.8±5.4	7.8±3.8	5.0±0.8
ZNF802sh-35	48.7	46.1	3.45	1.7	52.7±1.1	33.3±5.2	8.8±4.4	5.2±0.8
ZNF802sh-71	49.6	47.4	2.05	0.9	53.0±1.3	35.6±5.2	7.4±3.9	4.1±1.1

FigureS1. Globin chain analysis by reverse phase HPLC.

ZNF802

(JAZF1)

27 kDa

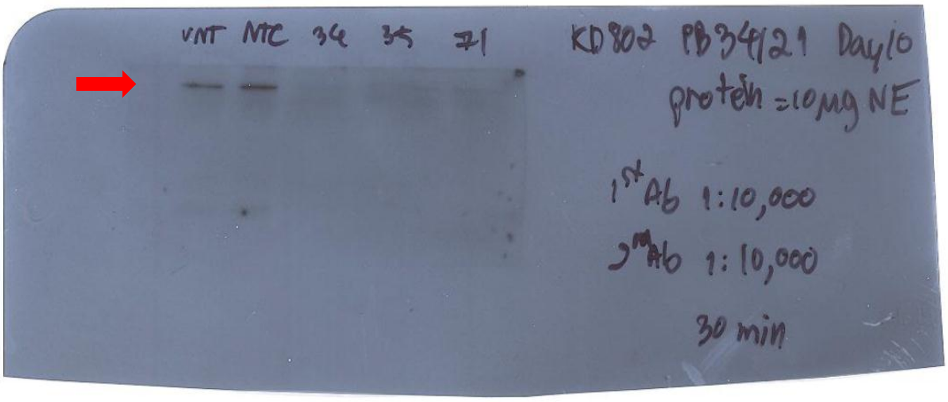

β-actin

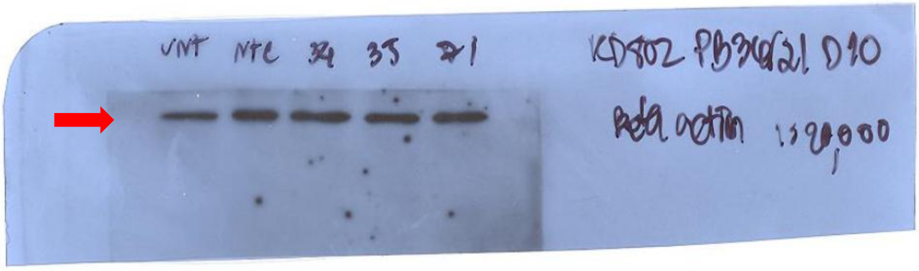

FigureS2. Western blot analysis to confirm knockdown ZNF802 in erythroblast.

A 10 μg of nuclear extract protein of untransduced (UNT) and transduced erythroblasts (shNTC, ZNF802sh-34, ZNF802sh-35, ZNF802sh-71) were run on 12% SDS-polyacrylamide gel and run with constant voltage 110V for 90 minutes in running buffer.

Western Blot : Globin protein in healthy donor

γ-globin

(HBG) 13 kDa

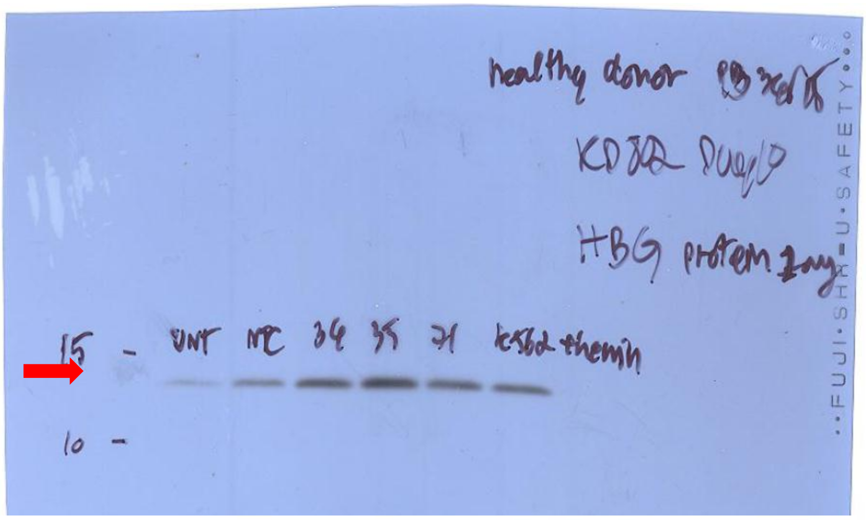

ε-globin

(HBE) 13 kDa

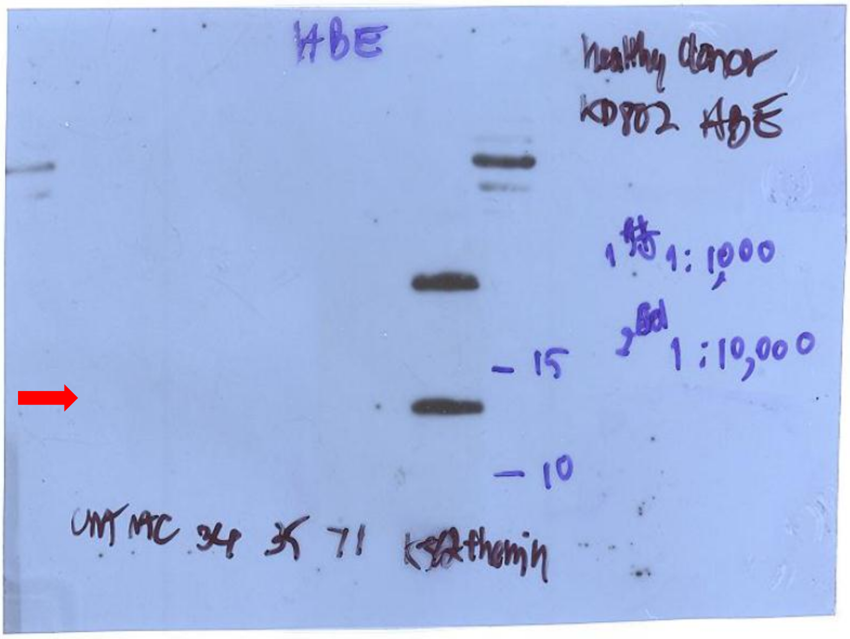

ζ-globin

(HBZ) 13 kDa

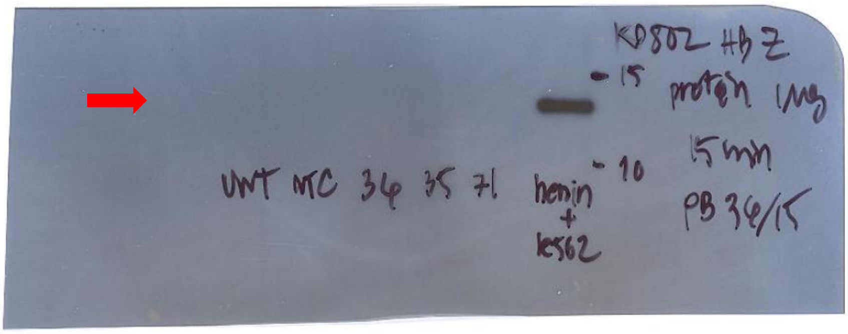

Western Blot: Globin protein in β^0-thalassemia/HbE

γ-globin

(HBG) 13 kDa

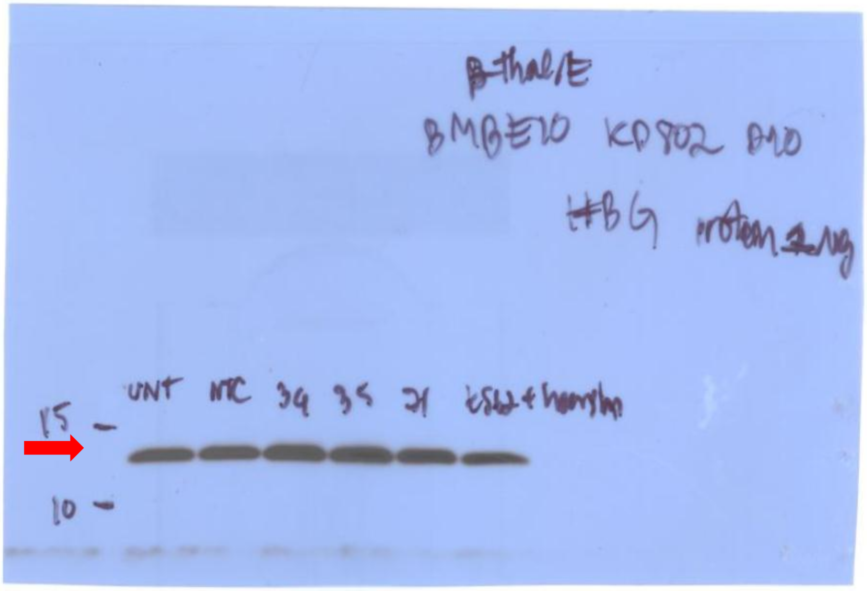

ε-globin

(HBE) 13 kDa

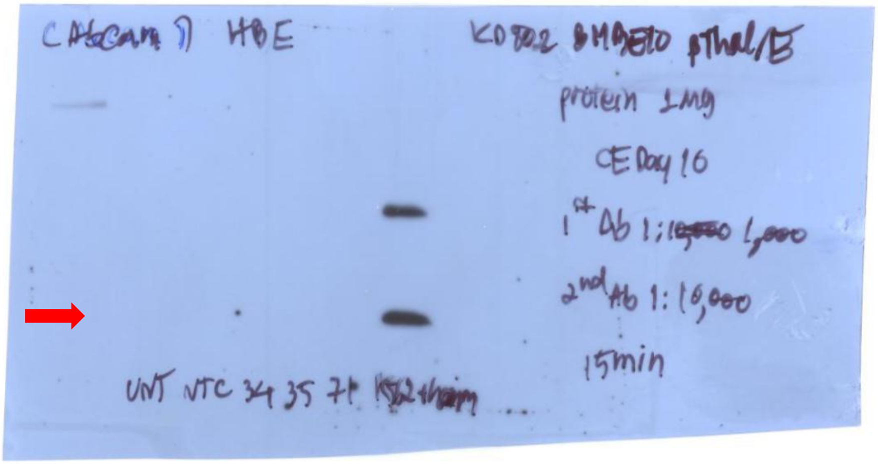

ζ-globin

(HBZ) 13 kDa

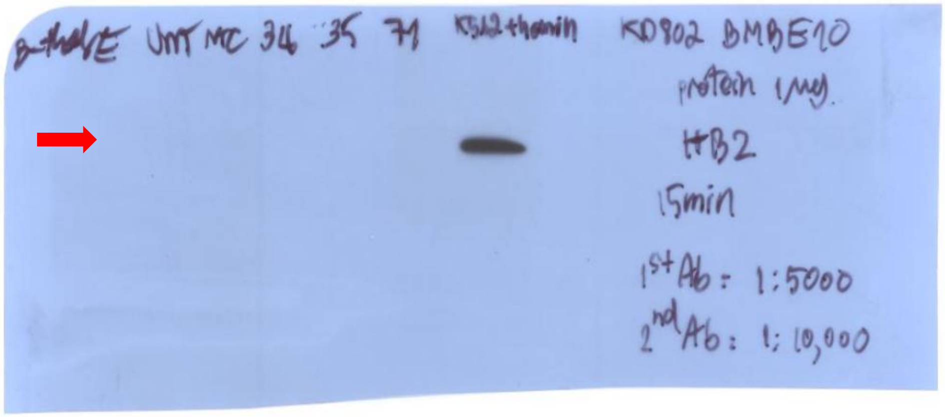

Loading control

β-actin

(healthy donor)

β-actin

(β^0-thalassemia/HbE)

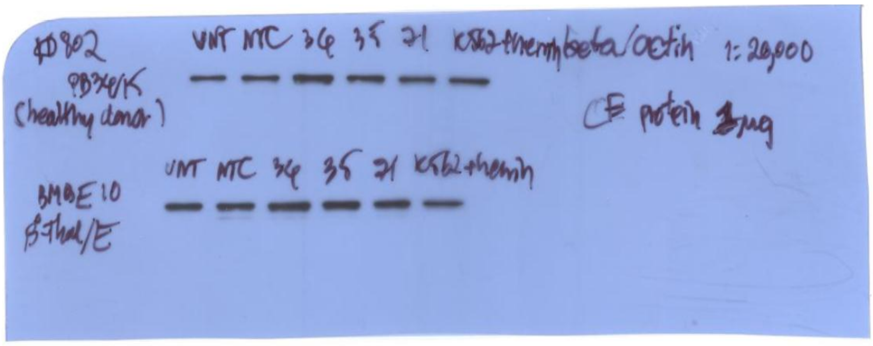

FigureS3. Western blots analysis to confirm globins protein expression post ZNF802 knockdown in erythroblast from healthy donor and β^0-thalassemia/HbE.

A 1 µg of cytosolic extract protein of untransduced (UNT), transduced erythroblasts (shNTC, ZNF802sh-34, ZNF802sh-35, ZNF802sh-71) and K562 treated with hemin (as positive control) were run on 12% SDS-polyacrylamide gel and run with constant voltage 110V for 90 minutes in running buffer.
